# Cytogenomic characterization of a de novo 4q34.1 deletion in a girl with mild dysmorphic features and a coagulation disorder

**DOI:** 10.1186/s13039-021-00564-z

**Published:** 2021-09-04

**Authors:** Juan Pablo Meza-Espinoza, José Alfredo Contreras-Gutiérrez, Eliakym Arámbula-Meraz, Juan Ramón González-García, Ma. Guadalupe Domínguez-Quezada, Noemí García-Magallanes, Jesús Madueña-Molina, Julio Benítez-Pascual, Miriam Partida-Pérez, Verónica Judith Picos-Cárdenas

**Affiliations:** 1 Facultad de Medicina e Ingeniería en Sistemas Computacionales de Matamoros, Universidad Autónoma de Tamaulipas, Matamoros, Tamps. Mexico; 2 Facultad de Medicina, Universidad Autónoma de Sinaloa, Culiacán, Sin. Mexico; 3 Laboratorio de Genética y Biología Molecular, Posgrado en Ciencias Biomédicas, Facultad de Ciencias Químico Biológicas, Universidad Autónoma de Sinaloa, Culiacán, Sin. Mexico; 4 División de Genética, Centro de Investigación Biomédica de Occidente, Instituto Mexicano del Seguro Social (IMSS), Guadalajara, Jalisco Mexico; 5 Laboratorio de Biomedicina y Biología Molecular, Unidad Académica de Ingeniería en Biotecnología, Universidad Politécnica de Sinaloa, Mazatlán, Sin. Mexico; 6 Facultad de Odontología, Universidad Autónoma de Sinaloa, Culiacán, Sin. Mexico; 7 Departamento de Ciencias Médicas, Centro Universitario de La Costa (CUCosta), Universidad de Guadalajara, Puerto Vallarta, Jalisco, México; 8 Laboratorio de Genética, Facultad de Medicina, Universidad Autónoma de Sinaloa, Culiacán, Sin. Mexico; 9 Servicio de Medicina Genética, Hospital General de Culiacán, Culiacán, Sin. Mexico; 10 Núcleo Académico Básico del Programa de Posgrado de la Facultad de Ciencias de la Nutrición y Gastronomía, Universidad Autónoma de Sinaloa, Culiacán, Sin. Mexico

**Keywords:** Chromosome 4, de novo 4q deletion, Cytogenomic characterization, aCGH, Clinical heterogeneity

## Abstract

**Background:**

4q deletion syndrome is a rare chromosomal disorder that mostly arises de novo*.* The syndrome is characterized by craniofacial dysmorphism, digital abnormalities, skeletal alterations, heart malformations, developmental delay, growth retardation, Pierre Robin sequence, autistic spectrum and attention deficit-hyperactivity disorder, although not every patient shows the same features. Array comparative genomic hybridization (aCGH) use improves the detection of tiny chromosomal deletions and allows for a better understanding of genotype–phenotype correlations in affected patients. We report the case of a 6-year-old female patient showing mild dysmorphic features, mild mental disabilities and a coagulation disorder as a consequence of a de novo del(4)(q34.1) characterized by aCGH.

**Case presentation:**

A 6-year-old female patient exhibited special craniofacial features, such as backward-rotated ears, upslanted palpebral fissures, broad nasal bridges, anteverted nares, broad nasal alae, smooth philtrums, smooth nasolabial folds, thin lips, horizontal labial commissures, and retrognathia. In the oral cavity, maxillary deformation, a high arched palate, agenesis of both mandibular canines and fusion of two mandibular incisors were observed. She also displayed bilateral implantation of the proximal thumbs, widely spaced nipples, dorsal kyphosis, hyperlordosis, and clitoral hypertrophy. In addition, the patient presented with coagulopathy, psychomotor delay, attention deficit-hyperactivity disorder, and mild mental disability. A chromosomal study showed the karyotype 46,XX,del(4)(q34.1), while an aCGH analysis revealed an 18.9 Mb deletion of a chromosome 4q subtelomeric region spanning 93 known genes.

**Conclusion:**

The clinical manifestations of this patient were similar to those reported in other individuals with 4q deletion syndrome. Although most of the patients with a 4q34 terminal deletion share similarities, variations in phenotype are also common. In general, clinical effects of chromosomal deletion syndromes depend on the length of the deleted chromosomal segment and, consequently, on the number of lost genes; however, in all of these syndromes, there is no simple correlation between the phenotype and the chromosomal region involved, particularly in cases of 4q deletion.

## Background

Chromosome 4q deletions are classified as interstitial and terminal. Interstitial deletions range from 4q11 to 4q28.3, and terminal deletions span from 4q31.1 [1, 2]. 4q deletion syndrome, due to either interstitial or terminal deletions, is an uncommon chromosomal disorder, with an incidence of nearly 1:100,000 [1], and most cases are de novo [3]. 4q deletions are diagnosed postnatally in equal proportions of males and females; in general, such deletions involve large segments and are detected by GTG banding (G-bands by trypsin and Giemsa) [3]. The principal clinical findings of 4q deletion syndrome are craniofacial dysmorphism (low-set ears, broad nasal bridge, short upturned nose, and micrognathia), digital anomalies, skeletal alterations, heart malformations, developmental delays, growth retardation, Pierre Robin sequence, autistic spectrum disorders and attention deficit-hyperactivity disorder [1, 3]. However, no patient shows all features [1]. Deletions of 4q33 to 4q35 are the least common, and generally, patients present with minor physical anomalies and mild mental disability [4]. Moreover, it has been challenging to determine the karyotype-phenotype correlation in each patient with chromosomal imbalances. However, the use of array comparative genomic hybridization (aCGH) improves the detection of subtle deletions and allows for a better understanding of genotype–phenotype correlations in affected patients [2, 5–9]. Here, we report the case of a 6-year-old female patient with mild dysmorphic features, mild mental disability, coagulopathies and a de novo del(4)(q34.1) deletion encompassing an 18.9 megabase (Mb) loss that includes 93 genes.

## Case presentation

**Fig. 1:**
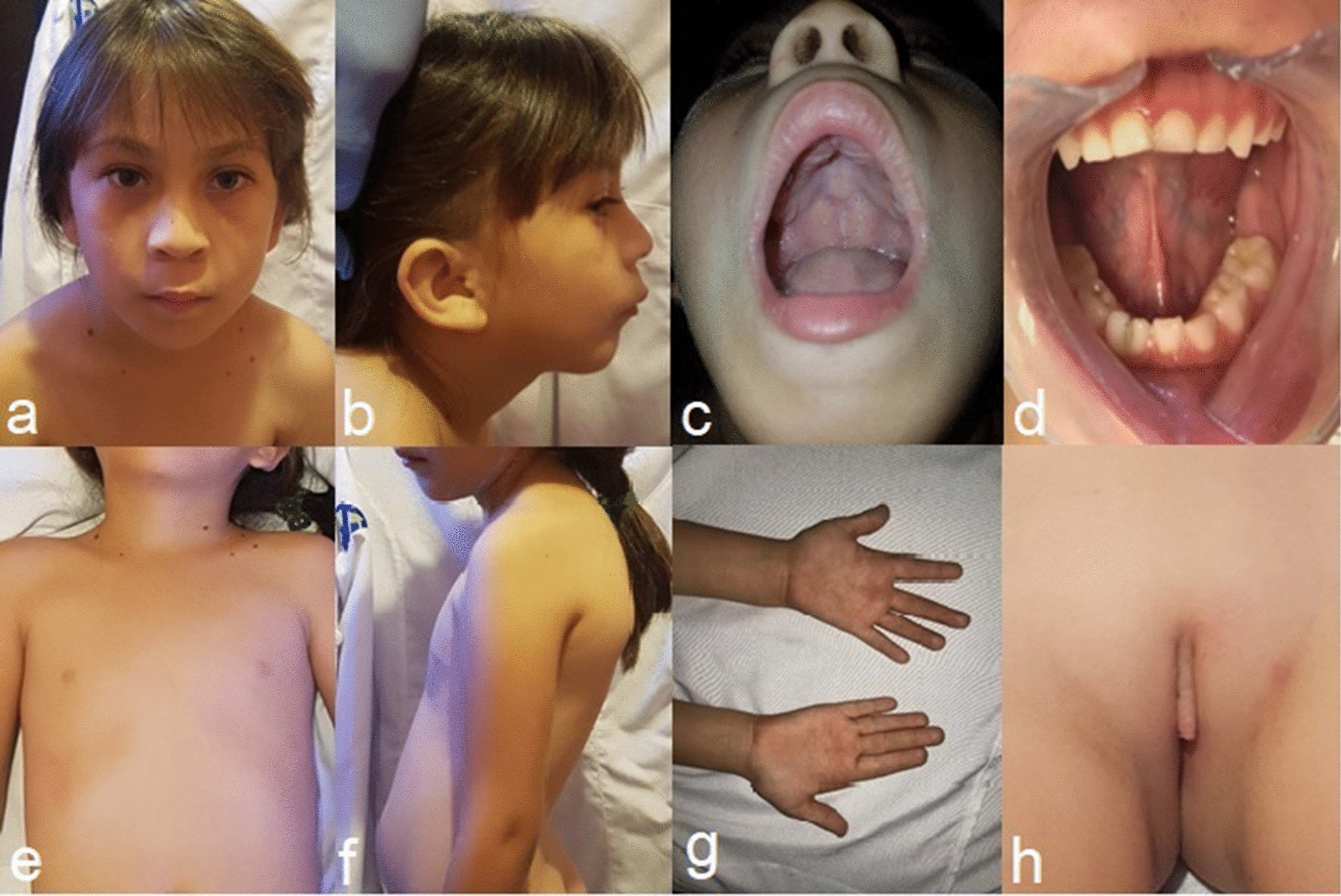
Clinical findings of the patient. **a** Upslanted palpebral fissures, broad nasal bridge, anteverted nares, broad nasal ala, smooth philtrum, smooth nasolabial folds, thin lips, horizontal labial commissures, and broad eyebrows. **b** Ears rotated backwards, retrognathia, and long eyelashes. **c** High arched palate. **d** Canines agenesis and fusion between the central and left lateral incisor of the mandible. **e** Nevi and widely spaced nipples. **f** Dorsal kyphosis and hyperlordosis. **g** Proximal implantation of the thumbs. **h** Clitoral hypertrophy

A 6-year-old female patient, the product of the third pregnancy of healthy nonconsanguineous parents (the mother was 20 years old, and the father was 29 at the time the patient was born), presented with poor suckling at birth due to facial muscle hypotonia. At the current physical examination, the patient presented with a height of 114 cm (50th centile), weight of 21.6 kg (50–75th centile), and head circumference of 52 cm (50–75th centile). Craniofacial features included backward-rotated ears, upslanted palpebral fissures, broad nasal bridge, anteverted nares, broad nasal alae, smooth philtrum, smooth nasolabial folds, thin lips, horizontal labial commissures, retrognathia, broad eyebrows, and long eyelashes (Fig. 1a, b). The oral cavity showed maxillary deformation, a high arched palate, agenesis of both canines of the mandible, and fusion between the central and left lateral incisors of the mandible (Fig. 1c, d). The patient had respiratory disorders due to nasal turbinate hypertrophy and hyperplastic tonsils (due to mouth breathing). Moreover, she had widely spaced nipples (Fig. 1e), dorsal kyphosis, hyperlordosis (Fig. 1f), and several nevi (less than 5 mm) in the left supraclavicular fossa, left clavicular region, and right and left hemicollar (Fig. 1e). Her hands showed proximal implantation of the thumbs and fetal fingertip pads (Fig. 1g). Her genitalia showed clitoral hypertrophy (20 mm) (Fig. 1h). Furthermore, the patient had coagulopathies presenting as recurrent hematomas and a long clotting time, as well as excessive bleeding during tooth loss. Her prothrombin time (PT) and activated partial thromboplastin time (aPTT) were 13 s and 44.5 s, respectively (reference values: 12–14 s and 35–43 s, respectively). Other anomalies included psychomotor delay, attention deficit-hyperactivity disorder, anxiety, sphincter control absence and mild mental disability.

## Results

**Fig. 2:**
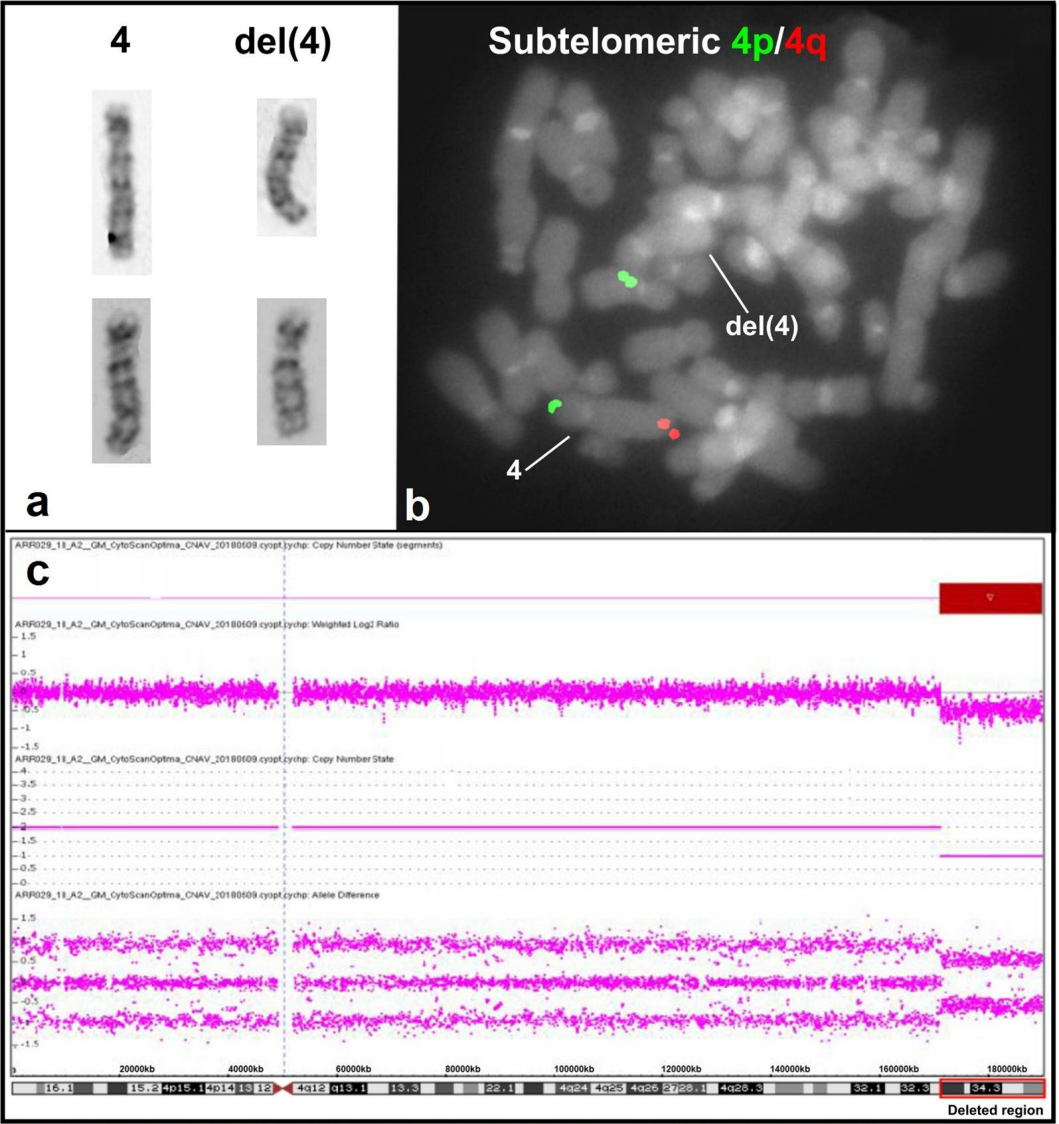
Chromosomal and aCGH studies in the patient. **a** Selected GTG-banded chromosomes. Normal chromosome 4 is on the left and chromosome 4 with the deletion is on the right. **b** FISH study with a mixture of subtelomeric 4p/4q molecular probes, labeled in green and red, respectively. The subtelomeric 4q red signal is absent from the deleted chromosome 4. **c** aCGH image exhibit an 18.9 Mb terminal deletion of the chromosome segment 4q34.1q35.2 (171,135,044-190,036,318, GRCh38/hg38). The deleted chromosomal region is delimited by the red rectangle

A chromosomal analysis of cultured peripheral blood lymphocytes from the patient was performed by the GTG-banding method at a resolution of 500–550 bands [10] and revealed a karyotype 46,XX,del(4)(q34.1) (Fig. 2a). Both parents were chromosomally normal. A FISH study with a mixture of commercial subtelomeric 4p/4q probes (Cytocell: LPT 04PG and LPT 04QR) confirmed the absence of one copy of subtelomeric 4q sequences on one chromosome 4 (Fig. 2b). To determine the genomic imbalance, aCGH on the proband was performed using CytoScan™ Technology (Thermo Fisher Scientific Inc). Reactions of digestion, ligation, PCR, purification of PCR products, quantification, fragmentation, labeling, matrix hybridization, washing, staining, and scanning arrays were performed according to the manufacturer’s instructions. Data were analyzed with ChAS 4.0 software. Interpretation of the results was performed using the following databases: Ensembl Resources, Database of Genomic Variants, Cytogenomics Array Group Copy Number Variant, Online Mendelian Inheritance in Man (OMIM), University of California Santa Cruz Database, ClinGen, and ClinVar databases. The analysis revealed the following 18.9 Mb deletion of a chromosome 4q region containing approximately 93 genes: arr[GRCh38] 4q34.1q35.2(171,135,044_190,036,318)×1 dn (Fig. 2c). Hence, the integrated proposita’s karyotype was 46,XX,del(4)(q34.1)[20].ish 4pter(subtel(4p)×2),4qter(subtel(4q)×1)[15].arr[GRCh38]4q34.1q35.2(171,135,044_190,036,318)×1 dn.

## Discussion and Conclusions

The patient’s net genomic imbalance was an 18.9 Mb deletion spanning approximately 93 known genes, from *MIR6082* to *FRG2,* including 44 genes recorded in the OMIM database. Among them, *HPGD, VEGFC, AGA, TENM3, TRAPPC11, CASP3, PRIMPOL, SLC25A4, UFSP2, TLR3, CYP4V2, DFNA24, PDLIM3, SORBS2, KLKB1, F11,* and *FRG1* are mutated in some disorders [11]. Other genes in this region, such as *HAND2, PDLIM3,* and *SORBS2,* have been implicated in congenital heart defects [2], particularly *HAND2* (chr4:173,526,091-173,530,229, GRCh38), of which mutations have been transmitted in a pattern of dominant inheritance [12].

Our patient presented with clinical features typical of 4q terminal deletions, as well as clitoral hypertrophy and idiopathic coagulopathy, but not cardiopathy. Vona et al. [2] reported the case of a young male patient with a 4q35.1q35.2 deletion (chr4:184,046,156-190,901,117, GRCh37; chr4:183,125,003-189,979,962, GRCh38) affecting 6.9 Mb, who, in addition to heart defects, hearing impairment, cleft palate, and bilateral cryptorchidism, at age five developed coagulation factor XI deficiency. Moreover, Guéguen et al. [13] reported the cases of a woman and two of her children with a 4q34.2 deletion spanning ~ 7 Mb (chr4:182,720,115-191,044,276, GRCh37; chr4:182,798,962-190,214,555, GRCh38) with a variable bleeding phenotype. These are the only known patients with 4q terminal deletion and a coagulation disorder, which could be related to the loss of two genes of the coagulation pathway, *KLKB1* (chr4:186,227,507-186,258,471, GRCh38) and *F11* (chr4:186,265,945-186,288,780, GRCh38), in particular *F11*, of which some mutations have shown dominant inheritance in patients with bleeding tendency [14]. To the best of our knowledge, clitoral hypertrophy has not been previously associated with 4q terminal deletions.

Several cases of terminal deletions overlapping 4q34 or 4q35 have been reported. Although many of the affected patients share similar clinical presentations, variations in phenotype are common. While our patient did not present with heart disease, she shares multiple features with these other patients, in particular facial dysmorphism, nasal anomalies, a high arched palate, digital anomalies, psychomotor delay, and mental disability (all these clinical features are summarized in Table 1) [4, 5, 7, 15–19]. In this regard, Rossi et al. [5] described a young woman with a sporadic 4q34.1q35.2 deletion encompassing 16.44 Mb (chr4:174,685,919-191,121,195, hg18; chr4:173,528,193-189,963,046, GRCh38, from genes *HAND2* to *FRG1*) who was diagnosed with learning disabilities, Pierre Robin sequence, and heart defects, such as atrial septal and Ebstein anomalies. A further de novo 4q34.1q35.2 deletion spanning 17.4 Mb (chr4:172,977,872-190,351,861, hg18; chr4:171,820,146-189,193,713, GRCh38) was detected in a young boy who presented with tetralogy of Fallot, right aortic arch, and facial dysmorphism, resembling 22q11.2 deletion syndrome [6]. Similarly, Tsai et al. [4] described a child with a de novo 4q34.2 terminal deletion who presented with cardiac defects, a cleft palate, learning difficulties, and right fifth finger anomalies, consistent with velocardiofacial syndrome, but negative for the 22q11.2 deletion. Connel et al. [20] reported a girl with 4q34 deletion who, in addition to facial dysmorphism, digital anomalies, and heart defects, also presented with bilateral optic disk swelling. Bendavid et al. [21] identified a woman and her daughter carrying a 4q34.3 terminal deletion distal to the *AGA gene* [at least from *TENM3* (chr4:182,243,402-182,803,024, GRCh38; approximately 8.7 Mb)] who exhibited different phenotypes. While the mother showed a cardiac defect, the daughter had congenital absence of the upper vagina and uterus. Marci et al. [22] described a female patient and her child carrying the same 4q34.3 deletion but with different phenotypes. While the mother had nonobstructive cor triatriatum sinister, the son presented with tetralogy of Fallot. Descartes et al. [18] reported a maternal 4q34.2 terminal deletion in two siblings with growth retardation, intellectual disability, and craniofacial alterations, but their mother only showed craniofacial alterations. Moreover, by aCGH studies, Youngs et al. [7] detected a 4q35.2 interstitial microdeletion of nearly 1.2 Mb (chr4:187.47-188.66, GRCh37; chr4:186,548,846-187,738,846, GRCh38), involving *MTNR1A*, *FAT1*, and *F11*, in an autistic boy who also showed congenital cardiopathy, psychomotor delay, facial dysmorphism, and mental disability. Similarly, through microarrays, Shao et al. [23] identified six patients with 4q35 deletion who had multiple congenital anomalies, with either dysmorphism, developmental delay/mental disability, or seizure disorders. They also reported two patients with 4q35 deletion and minor alterations. One had only dysmorphic features, while the other showed respiratory distress syndrome and asthma exacerbation. Strehle et al. [9] studied a patient with almost all clinical characteristics of 4q deletion syndrome and found a deletion of nearly 465 kb in 4q35.1 (chr4: 186,770,069-187,234,800, hg18; chr4:185,611,921-186,076,652, GRCh38, case 20); they proposed that this region is critical for the expression of this condition. Nevertheless, 4q35 terminal deletions have also been found in patients who only have autism or psychiatric diseases. Chien et al. [8] described a boy with autism and 4q35.1q35.2 deletion affecting approximately 6.8 Mb (chr4:183,904,000-190,720,000, hg18; chr4:182,745,853-189,561,852, GRCh38), and Pickard et al. [24] identified a man with a schizoaffective disorder and mental disability who had a 4q35.2 deletion, spanning approximately 3.0 Mb (nearly chr4:187,214,555-190,214,555, GRCh38). Additionally, several individuals with 4q34 or 4q35 deletions and a normal phenotype have been reported [13, 25–29, Table 2].

**Table 1:** Clinical findings in patients with deletions of the chromosome 4q34qter and 4q35qter

Reference	Present case	Lin et al. [15] case 4	Vogt et al. [16] case 4	González et al. [17]	Rossi et al. [5] case 2	Descartes et al. [18]	Descartes et al. [18]	Caliebe et al. [19]	Tsai et al. [4]	Vona et al. [2]	Youngs et al. [7]
Deletion	4q34.1	4q34	4q34	4q34	4q34.1	4q34.2	4q34.2	4q34.2	4q34.2	4q35.1q35.2	4q35.2
Reported assembly		ND	*HAND2*	ND	hg18 174,685,919-191,121,195	ND	ND	ND	ND	GRCh37/hg19 184,020,463-190,993,669	GRCh37/hg19 187,470,000-188,660,000
GRCh38/hg38	171,135,044-190,036,318				173,528,193-189,963,046					183,099,257-190,075,799	186,548,846-187,738,846
Etiology	de novo	ND	ND	ND	de novo	mat	mat	de novo	de novo	de novo	ND
Sex	F	M	^**a**^F	M	F	^**b**^M	^**b**^M	M	^**c**^M	^**d**^M	M
Loss (Mb)	18.9	ND	ND	ND	16.4	ND	ND	ND	ND	6.9	1.2
Facial dysmorphism	+	+	−	+	+	+	+	+	−	+	+
Micro/retrognatia	+	−	−	−	+	−	−	−	−	−	−
Prominent forehead	−	+	−	+	−	+	+	−	−	−	−
UPF	+	+	−	−	−	+	−	−	−	−	−
Nistagmus	−	+	−	−	+	−	+	−	−	−	−
PR/LE	+	−	−	−	−	−	+	−	−	−	−
Nasal anomalies	+	+	−	−	+	+	+	+	+	−	+
Thin lips	+	−	−	−	−	+	+	+	−	−	+
High palate	+	+	−	−	−	+	+	−	+	+	+
Digital anomalies	+	+	+	+	+	−	−	−	+	−	+
Heart anomalies	−	−	+	−	+	−	−	−	+	+	+
CD/Hemorrhages	+	−	+	−	−	−	−	−	−	+	−
GA	+	−	−	+	+	−	−	+	−	+	−
Growth retardation	−	+	−	−	+	+	−	−	−	−	−
Psychomotor delay	+	+	+	+	+	+	−	+	+	+	+
Mental disability	+	+	−	+	+	−	+	+	+	−	+
PRS	−	−	−	−	+	−	−	−	−	−	−

*F* female, *M* male, *UPF* upslanted palpebral fissures, *PR/LE* Posteriorly rotated/low ears. *CD* Coagulation disorders. *GA* genitourinary anomalies. *PRS* Pierre Robin sequence. *ND* not determined

^a^The patient presented tracheal hemorrhages

^b^These patients are siblings; their mother, carrier of the same deletion showed prognatism, nystagmus, PR/LE, nasal anomalies, thin lips, and high palate

^c^He also exhibited bifid uvula

^d^The patient had deficiency of coagulation factor XI (48%), elevated prothrombin time, and bifid uvula. + Denotes the presence, whereas − denotes absence of a characteristic

*Note*: If the authors did not report additional clinical characteristics, we considered them were absent

**Table 2:** Individuals with 4q34 or 4q35 deletions and normal phenotype

Karyotype	Reported assembly: GRCh37/hg19	GRCh38/hg38 assembly	Genomic loss	References
^a^46,XX,del(4)(q34.2)	chr4:182,720,115-191,044,276	chr4:182,798,962-190,214,555	~ 7 Mb	[13]
^b^46,XX,del(4)(q34.1q34.3)	chr4:173,004,000-182,313,000	chr4:172,082,849-181,391,847	9.3 Mb	[25]
^c^46,XX,del(4)(q35.1q35.2)	chr4:184,717,878-190,469,337	chr4:183,796,725-189,548,183	5.75 Mb	[26]
^d^46,XX,del(4)(q35)	ND	ND	1.15–1.3 Mb	[27]
^e^46,XY,del(4)(q34.2)	ND	ND	ND	[28]
^f^46,XY	ND	ND	4q subtelomere deletion	[29]
^g^del(4)(q34.1q34.2)	ND	ND	ND	

^a^This female and two of her children (also carriers of the deletion) showed only variable bleeding phenotype

^b^This patient had three consecutive miscarriages

^c^Karyotype detected in a female and her two daughters

^d^This patient had two children with mental disability

^e^This male had a girl who died perinatally due to congenital heart defects

^f^This patient had a girl with mental disability, developmental delay, upper palpebral fissures, chorea movements, and fifth finger clinodactyly, and a child with mental disability and developmental delay (cases 60 and 61, respectively)

^g^This case was registered from http://cs-tl.de/DB/CA/HCM/4-HM.html#3, where the sex was not specified. ND: Not determined

Interestingly, it should be noted that although Pierre Robin sequence is commonly associated with 4q deletion, only one patient in our review showed this feature, which could indicate that 4q34 → qter is not a critical region for Pierre Robin sequence. Since not every individual with deletions involving the *HAND2* gene presents with congenital heart defects (neither our patient nor the patient described by Huang et al. [28] presented with such defects) and because some patients with distal deletions of *HAND2* do show those defects [2, 4, 5, 7, 21, 22, 28], it is probable that other genes in this region play important roles in heart development, as is the case for the *SORBS2* gene [30], located at 4q35.1. Last, although clinical effects generally depend on the length of the chromosomal segment and, consequently, on the number of genes deleted, there is no direct correlation between the phenotype and the chromosomal region involved. Even though the reasons why similar deletions result in great phenotypical heterogeneity or even in normal phenotypes are not currently known, environmental variables interacting with the genetic background, some modifier genetic variants and/or epigenetic changes could play key roles that contribute to such heterogeneity [31]. It is obvious that further research is necessary to clarify this issue.

## Data Availability

Data generated or analyzed during this study are included in this published article.

## Abbreviations

aCGH: Array comparative genomic hybridization
Mb: Megabase
GTG-banding: G-bands by trypsin using Giemsa
PT: Prothrombin time
aPTT: Activated partial thromboplastin time
